# From molecules to neural morphology: understanding neuroinflammation in autism spectrum condition

**DOI:** 10.1186/s13229-016-0068-x

**Published:** 2016-01-20

**Authors:** Adam M. H. Young, Bhismadev Chakrabarti, David Roberts, Meng-Chuan Lai, John Suckling, Simon Baron-Cohen

**Affiliations:** Autism Research Centre, Department of Psychiatry, University of Cambridge, Douglas House, 18B Trumpington Road, Cambridge, UK; School of Clinical Medicine, Addenbrookes Hospital, University of Cambridge, Cambridge, UK; Centre for Integrative Neuroscience and Neurodynamics, School of Psychology and Clinical Language Science, University of Reading, Reading, UK; Centre for Addiction and Mental Health and Department of Psychiatry, University of Toronto, Toronto, Canada; Department of Psychiatry, National Taiwan University Hospital and College of Medicine, Taipei, Taiwan; Brain Mapping Unit, Department of Psychiatry, University of Cambridge, Cambridge, UK; Behavioural and Clinical Neuroscience Institute, University of Cambridge, Cambridge, UK; CLASS Clinic, Cambridgeshire and Peterborough NHS Foundation Trust, Cambridge, UK

**Keywords:** NF-κB, Autism, Brain, Inflammation

## Abstract

Growing evidence points toward a critical role for early (prenatal) atypical neurodevelopmental processes in the aetiology of autism spectrum condition (ASC). One such process that could impact early neural development is inflammation. We review the evidence for atypical expression of molecular markers in the amniotic fluid, serum, cerebrospinal fluid (CSF), and the brain parenchyma that suggest a role for inflammation in the emergence of ASC. This is complemented with a number of neuroimaging and neuropathological studies describing microglial activation. Implications for treatment are discussed.

## Background

The presence of inflammation in autism spectrum condition (ASC) is a concept that is gathering momentum. Traditionally, different forms of ASC have been recognised: Classic (or Kanner type) autism (which can entail general intellectual disability and language delay), Asperger syndrome (which entails no developmental language delay or intellectual disability) and syndromic forms of autism such as co-occurring with Rett syndrome, Fragile X syndrome, tuberous sclerosis complex and Timothy’s syndrome, to name a few [29]. In DSM-5, these are now subsumed under a single umbrella term of autism spectrum disorder (ASD). In this article, we use the term autism spectrum condition (ASC) because the term ‘disorder’ is regarded by some as stigmatising and the term ‘condition’ acknowledges both the disability and the differences and strengths in such individuals.

The key behavioural features defining ASC are the presence of difficulties in social reciprocity and communication, alongside unusually narrow interests, repetitive behaviours and speech, insistence on sameness, and idiosyncratic sensory responses DSM-V® [30]. Cognitively, ASC is described as a condition characterised by weakened central coherence [35, 46], executive dysfunction [76], and mentalising difficulties [5, 6] alongside strengths in ‘systemizing’ [5] and attention to detail [82, 83]. Genetic, environmental, neurological, and immunological factors contribute to its aetiology [72].

Some, but not all, studies suggest that ASC involves early brain overgrowth [21, 74]. This is unlikely to be a universal phenomenon [17, 73] and is one of the key factors that could be linked to the heterogeneity of the condition together with neuroinflammation. Nevertheless, there are a multitude of developmental reasons which could be responsible for this observation, including atypical pruning of synapses [41] and, more recently, neuron density has been shown to play a significant role [22], but this could also reflect neuroinflammation. Animal models have identified microglial priming as a major factor in a causal chain that leads to the wide spectrum of neuronal dysfunctions and behavioural phenotypes [59]. Increased head size in people with ASC correlates positively with a history of allergic/immune disorders [86]. In particular, there is an association between ASC and neuroinflammation in anterior regions of the neocortex [79, 101, 110], resulting from activation of microglia and astrocytes [2]. Gene networks involved in immune processes are overexpressed in the brain of individuals with ASC [102, 103]. This has been linked to atypical expression of nuclear factor kappa-light-chain-enhancer of activated B cells (NF-κB) in a number of cell types in ASC including neurons, astrocytes, and microglia [107]. This has a linear relationship with pH changes localised to the lysosomal compartments of mature microglia [107], indicating increased immune activity. Microglia play a critical role in the pruning of synapses, thus providing a potential bridge between the atypical synaptic pruning and the immune dysregulatory hypotheses of ASC [78]. Below we review the mechanisms that may underlie neuroinflammation and the evidence at genetic and protein levels for each of these mechanisms (Table 1).

**Table 1:** Summary of contributing immunological factors in ASC

Level of biological processes	Immunological factors involved
Genetic	IFN-γ, Toll-like receptors, T-cell receptor
Extracellular mediators	Maternal antibodies, cytokines and chemokines
Cell surface proteins	Human leukocyte antigen, Toll-like receptors
Intracellular signalling	mTOR, PTEN, NF-κB
Neural changes	Neuron density, glial proliferation

### Genetic studies

Many loci have been implicated in the condition [42]. Most recently, a number of recent breakthroughs have dramatically advanced our understanding of ASC from the standpoint of human genetics and neuropathology. These studies highlight the period of foetal development and the processes of chromatin structure, synaptic function, and neuron-glial signalling [18]. A key component of genetic architecture is the allelic spectrum influencing trait variability. Recent studies have demonstrated that the total heritability of risk-associated genes is in the range of 50–60 % with common variants explaining the bulk of it [40]. In a comparison of genome-wide linkage studies between autoimmune and inflammatory disorders in ASC, overlap of polymorphic markers were statistically significant [8]. For a comprehensive list of the genes identified, see http://www.grc.nia.nih.gov/branches/rrb/dna/atsmap.htm.

Where it is conceded that the chromosomal regions identified in these linkage studies and the specific variants of genes identified in genetic association studies are quite often not unique to any one disorder [8]. Specific immunological genetic assays have been tested in panelled assays to test both sensitivity and specificity in diagnosing autism. Using a signature of differentially co-expressed genes that were enriched in translation and immune/inflammation function, the authors were able to identify boys with autism with 83 % accuracy [85].

### Extracellular mediators

#### Maternal-foetal transfer

The involvement of an inflammatory pathogenesis in ASC likely originates during the gestational period [59]. Autoantibodies are transferred from the mother to the child during pregnancy and are associated with a number of factors that affect both pregnancy and neonatal outcome [100]. In a cohort of mothers of children with ASC, autoantibodies have been detected against critical neuronal components of foetal brain tissue samples [109] as well as transfer of maternal neuro-specific proteins [23]. These studies identified a range of unknown protein targets ranging from 30 to 250 kilodaltons (kDa) in size. Consecutive independent studies have identified autoantibodies that bind to novel proteins of 37, 39, and 73 kDa in size [39, 104].

A number of models have been hypothesised to explain the transfer of anti-foetal brain autoantibodies [58]. In one study, immunoglobulin G (IgG) isolated from mothers with children with ASC was transferred into rhesus macaque monkeys during mid-gestation and resulted in distinct behavioural changes in the offspring. In particular, the monkeys spent significantly less time in contact with their peers and spent more time in a non-social state. This was attributed to the specific IgG from mothers of children with ASC infusion and not observed in monkeys receiving IgG from control donors or monkeys that were saline treated [7, 65]. These observations have been replicated in mice [15, 93]. Whether the transfer of these auto-antibodies during gestation plays a role in the pathogenesis in ASC remains uncertain; however, it is clear that there is a potential association. Brimberg et al. [13] describes mothers of an ASC child being found to be four times more likely to harbour anti-brain antibodies than other women of child-bearing age. Fundamentally, these processes may have a significant impact on neurodevelopment. Supporting this view is a recent study from Braunschweig et al. [11] who have identified that lactate dehydrogenases A and B (LDH), cypin, stress-induced phosphoprotein 1 (STIP1), collapsin response mediator proteins 1 and 2 (CRMP1, CRMP2), and Y-box-binding protein comprise the seven primary antigens of maternal autoantibody-related (MAR) autism. This built on previous work which introduced the concept of studying the maternal plasma antibodies against both the maternal and fatal brain [12, 24, 87].

A number of animal studies have demonstrated that placenta to foetus transport can alter development. Specifically, Lin et al. [63] used *P. gingivalis* in mice to demonstrate that maternal immune system stimulation can lead to elevated levels of pro-inflammatory cytokines in both the placenta and amniotic fluid, whilst at the same time decreasing the major anti-inflammatory cytokines TGF-β, IL-4, and IL-10. Maternal inoculation with Poly(I:C), as well as lipopolysaccharide (LPS), also in mice, have resulted in the animal displaying behavioural characteristics in keeping with ASC, including pre-pulse inhibition deficits, working memory deficits, and social interaction deficits [80]. Blocking the action of these pro-inflammatory cytokines during maternal infection was observed to inhibit the development of such behaviour [52, 80]. Maternal LPS administration upregulates both tumour necrosis factor-alpha (TNF-α) and IL-1β mRNA expression in the foetuses of pregnant rats in a dose-dependent manner [38].

#### Cytokines and chemokines

Cytokines and chemokines are pleiotrophic proteins that coordinate the host response to infection as well as mediate normal, ongoing communication between cells of non-immune tissues, including the nervous system [27]. As a consequence of this dual role, cytokines induced in response to an adverse stimuli (i.e. maternal infection or prenatal hypoxia) can profoundly impact fetal neurodevelopment. Aberrant levels of proinflammatory cytokines, interleukin 6 (IL-6), TNF-α and monocyte chemotactic protein-1 (MCP-1), not only in brain specimens and cerebrospinal fluid (CSF; [90, 101]) but also in amniotic fluid [1], index an active inflammatory process both in children and adults with ASC. These molecules act to increase immune cell recruitment and proliferation. Immune pathways are activated by proinflammatory cytokines such as TNF-α and IL-6 that stimulate the nuclear translocation of various transcription factors, including NF-κB that subsequently results in the potentiation of the immune response [81]. This is tightly controlled in acute infection and lasts for a limited time. However, the presence of such molecules in the absence of an acute stimulus is an atypical response. An atypical inflammatory response has been observed in peripheral samples to show similar changes [56] as well as decreases in anti-inflammatory protein IL-10 [56]. In a larger multi-analyte profiling (MAP) analysis, Suzuki et al. [98] reported from a total of 48 analytes examined, the plasma concentrations of IL-1β, IL-1RA, IL-5, IL-8, IL-12(p70), IL-13, IL-17, and growth regulated oncogene-alpha (GRO-α) were significantly higher in individuals with ASC compared with the corresponding values of matched controls, after correction for multiple comparisons. Upregulation of inflammation-related molecules has also been found to be characteristic for adult males (but not females) with Asperger syndrome [95]. In mid-gestation maternal serum, elevated concentrations of IFN-γ, IL-4, and IL-5 were significantly associated with a 50 % increased risk of ASC, regardless of ASC onset type and the presence of intellectual disability [45].

The main issue surrounding the reporting of serum results is that they show considerable within- and between-group variability. As such, the subtle differences found may indicate the presence of separate subgroups of the condition [60]. For example, statistical clustering analysis on large-scale clinical data suggests the presence of subgroups with ASC characterised by co-occurrence of infectious disorder [31], which could be related to physiological atypicality related to inflammatory processes. Further analysis using appropriately powered studies will be required in order to gauge the potential explanatory power of this hypothesis.

### Cell surface proteins

In contrast to cytokines and chemokines, major histocompatibility complex (MHC) family members have very short intracellular domains not thought to function in intracellular signalling cascades, but instead by interacting with a variety of receptors during cell-mediated immunity [92]. Together with TLRs, they form a key role in activity-dependent brain development and plasticity as well as regulating the immune response [84]. Specifically, it has been observed that (MHC) class I molecule H2-D(b) is essential for synapse elimination in the retinogeniculate system [62, 67].

Genetically, immune dysfunction in ASC has been suggested to included the MHC region, as this is an immunologic gene cluster whose gene products are class I, II, and III molecules. Class I and II molecules are associated with antigen presentation. The human leukocyte antigen (HLA) genes are among the strongest predictors of risk for autoimmune conditions. Some studies have observed that different HLA haplotypes are associated with neurodevelopmental conditions such as ASC [19, 55] and schizophrenia [94]. Stubbs et al. [97] initially demonstrated that mothers of children with ASC share HLA haplotypes with their children more often than in non-affected pairs. Recent evidence has suggested that impairments of innate immunity, originating with cell surface proteins, may play an important role in ASC. Enstrom et al. [33] demonstrated an improved responsiveness to signalling via select TLRs: TLR 2, TLR 4, and conversely a decreased production of cytokines following stimulation of TLR 9.

### Intracellular signalling pathways

Another distinct pathway to be implicated in ASC is a well-known family of transcription factors, the nuclear factor kappa B (NF-κB), which is one of the key players in the regulation of inflammatory responses [77, 81]. This transcription factor is constitutively expressed in the cytoplasm and is inhibited by inhibitor κB (I κB), which binds NF-κB, masking its nuclear localization signal and retaining it in the cytoplasm [44]. NF-κB activity is attributed to Rel/NF-κB family proteins forming homodimers and heterodimers through the combination of the subunits p65 (or RelA), p50, p52, c-Rel, or RelB [81]. Cytokines, chemokines, and reactive oxygen species are among a number of key mediators that induce NF-κB by activating IκB kinases [77]. These phosphorylate IκBα, leading to its polyu biquitination and degradation [43], allow NF-κB to migrate to the nucleus, where it activates the transcription of various proinflammatory genes.

NF-κB has been found to be aberrantly expressed in the orbitofrontal cortex in postmortem studies of adults with ASC [107]. Expression was most abundant in microglia followed by astrocytes compared to neurons. However, samples from other frontal lobe or cerebellar samples have not shown similar increases [64] suggesting a localisation of aberrant expression. Peripherally, NF-κB was upregulated in TNF-α and LPS stimulated peripheral blood monocytes (PBMCs) [71]. The upregulation of expression was found to be similar in that Young et al. [107] reported a 2.9-fold increase in NF-κB expression centrally compared to a 2.2-fold increase reported peripherally [71]. The significance of this similarity is difficult to ascertain as there is significant heterogeneity of brain parenchyma in response to inflammatory stimuli [108]. The implication of the NF-κB signalling pathway in ASC further supports a potential role for neuroinflammation. Further work is needed to identify whether upregulation of NF-κB plays a role in initiating the signalling cascade or whether it is a result of aberrant stimulation.

### Neuroanatomical changes

The ultrastructural morphology in ASC has been described as having atypical microglial and astroglial activation [101]. Prominent histological changes have been described in the cerebellum, characterised by a patchy loss of neurons in the Purkinje cell layer (PCL) and granular cell layer (GCL) [101] as well as a reduction in neuron number in the amygdalae and fusiform gyrus [89]. Glial activation has been widely observed throughout a number of independent studies. Vargas et al. [101] observed an increase in glial fibrillary acidic protein (GFAP) concentration in the white matter of the middle frontal gyrus (MFG) and anterior cingulate gyrus (ACG). An increased ratio of CD11c-positive, mature (highly active) microglia was observed in the orbitofrontal cortex and showed strong correlation with cell signalling molecules [107]. Microglial activation and increased microglial density was also observed in the dorsolateral prefrontal cortex in ASC [69]. Morphological alterations included somal enlargement, process retraction and thickening, and extension of filopodia from processes [69]. They also described a significant increase in microglial somal volume in the white matter. Microglial cell density was increased in the grey matter with non-significant trends in somal volume [69]. A recent study by Paolicelli and Gross [78] suggests a central role for microglia in synaptic pruning, a process that has been suggested to be aberrant in the developing brain in ASC [22].

Magnetic resonance spectroscopy (MRS) has provided significant insight into the ultrastructural morphology in ASC. Myo-inositol (Ins) is a metabolic compound located mostly in astrocytes. High Ins levels are thought to indicate an increase in astrocyte populations and are particularly abundant in neuroinflammation [10]. Interestingly, increased Ins levels have been demonstrated to impact on performance IQ scores in individuals with ASC [37].

The resonance group attributed to the glutamine-glutamate-GABA complex (Glx) includes contributions from both glutamate and its precursor glutamine. Very little glutamate penetrates the blood–brain barrier, so local synthesis is essential. De novo synthesis is mediated mainly by astrocytes, and as such, in vivo levels can be altered in neuroinflammation [61]. Glutamate synthesised by astrocytes is converted to glutamine via the enzyme glutamine synthetase and exported to neurons via multiple transporters [26]. In neurons, glutamine is reconverted into glutamate using the mitochondrial enzyme phosphate-activated glutaminase and then packaged into synaptic vesicles for release [61]. Release of glutamate initiates signalling events in excitatory neurotransmission; transmission is terminated by the removal of glutamate from the extracellular space, predominantly via astrocytic glutamate transporters. This overall cycle of synthesis, release, and recovery of glutamate is referred to as the glutamate–glutamine cycle [50].

DeVito et al. [28] described widespread decrease in N-acetylaspartate (NAA) and Glx among people with ASC, with reductions observed in both the cerebral grey matter and the cerebellum. In a sample of adults with ASC, there was also a significant decrease in concentration of Glx, as well as choline, creatine (Cr) and NAA, in the basal ganglia [51]. A reduction in Glx would classically be attributed to a reduction in neuron density. Nevertheless, inflammatory molecules can also contribute to inhibiting the astrocytic glutamate reuptake [53] and by inducing changes in glutamate receptor subunit expression, thus leading to reduced intracellular levels of Glx [4].

### Specificity of neuroinflammation

Neuroinflammation is emerging as a common finding in neurological and neuropsychiatric phenotypes. Most recently, it emerged as a finding in large genetic analyses of schizophrenia, bipolar disorder, and major depression [75]. This prompts the question of the specificity of inflammation as a contributing mechanism in the emergence of ASC. Furthermore, it promotes ongoing discussion on whether this is a causal or reactive process. What is certain is that, regardless of whether inflammation is specific to a condition or general to a majority of psychiatric conditions, if it is present, it should be treated and whether it forms part of a multisystme pathology, it should be treated and the response evaluated to observe whether there is a significant cognitive change. Clarifying the roles of various neuroinflammatory processes, causal (aetiological) or reactive, in neurodevelopmental conditions, also serves as a useful angle to delineate ubiquitous pathogenetic processes across all kinds of atypical neurodevelopment from specific mechanisms or reactive processes that mark one clinical diagnosis but not others.

### Evidence against inflammation as a contributing mechanism in autism

There have been a few studies that failed to identify atypical inflammatory activity in ASC [64, 102]. Voineagu et al. [102] detail that the immune changes observed in their study of convergent molecular pathology have a less pronounced genetic component and thus are most likely either secondary phenomena or caused by environmental factors. Some have even gone as far to say that there is evidence that the immune response is not overactivated [64]. Whilst these studies are important, they have not been replicated to the extent of papers supporting a role for inflammation in the pathogenesis of ASC. It is also important to consider that the scale of the inflammatory response is so vast that it is possible to target inflammatory mediators which may not contribute to the condition. This is well presented in a recent meta-analysis looking at the cytokine response in autism [66]. Where 19 cytokines were assessed, only 7 were observed to have significantly different levels in ASC.

## Conclusions

An emerging focus of research into the aetiology of ASC has suggested neuroinflammation as one candidate underlying biological model. With over 1000 candidate genes associated with it, ASC has a strong genetic component [70]. Similar to most medical conditions, however, there is also a significant environmental component in place, through gene-environmental interplay such as epigenetic mechanisms. The study of inflammation in ASC provides an excellent opportunity to dissect potential gene-environmental interplay. Here, we have highlighted some of the common indications of immunological dysregulation as potential contributing pathogenic processes for, at least certain subgroups of, individuals with ASC. Nevertheless, it is unclear whether the role of inflammation in ASC induces epigenetic change, via the activation of signalling cascades, or whether it is a direct result of genetic mutation and downstream effects. Whilst there is a growing body of work to support the role of inflammation in ASC, the greatest area of weakness in the field is that in general, the findings tend to be from individual studies and rarely are these replicated. Future work is needed to demonstrate what are essentially preliminary findings in large-scale studies. How much of a role, if any, neuroinflammation has on the emergence of ASC and contributing to its aetiological heterogeneity remains to be clarified.

## Abbreviations

ASC: autism spectrum condition
NF-κB: nuclear factor kappa-light-chain enhancer of activated B cells
